# Mental illness severity and characteristics among holocaust survivor immigrants, Non-Holocaust immigrants, and native israelis: A historical prospective study

**DOI:** 10.1007/s00127-025-02979-0

**Published:** 2025-08-21

**Authors:** Shikma Keller, Bella Savitsky, Orly Manor, Uriel Heresco-Levy, Pesach Lichtenberg

**Affiliations:** 1 Department of Psychiatry, Hadassah Medical Center Hebrew University of Jerusalem, Jerusalem, Israel; 2 Department of Nursing, School of Health Sciences, Ashkelon Academic College, Ashkelon, Israel; 3 The Hebrew University-Hadassah, Braun School of Public Health, Jerusalem, Israel; 4 Faculty of Medicine, The Hebrew University of Jerusalem, Jerusalem, Israel; 5 Department of Psychiatry, Herzog Memorial Hospital, Jerusalem, Israel; 6 Department of Psychiatry, Jerusalem Mental Health Center, Jerusalem, Israel

**Keywords:** Mental illness, Holocaust, Immigration, Genocide, Post-traumatic stress, Psychiatric hospitalization, War, Trauma, Childhood trauma

## Abstract

**Introduction:**

Holocaust survivors were exposed to extreme trauma. More than half a million survivors immigrated to Israel over the years, as well as immigrants who didn’t suffer the atrocities of the holocaust. Trauma and immigration are both risk factors for mental disorders.

**Aim:**

To describe differences in hospitalization characteristics and to determine whether there are differences in illness severity between mentally ill Holocaust Survivor Immigrants (HSI), non-Holocaust immigrants (NHI), and Native Israelis (NI).

**Methods:**

An unidentified list of hospitalized psychiatric patients was extracted from the Israel psychiatric case registry according to the following criteria: Jewish patients who were born in Europe or Israel before 1944 and were admitted to a psychiatric ward between 1945 and 2010. 30,539 records were divided into three groups: Holocaust Survivor Immigrants, Native Israelis, and Non-Holocaust Immigrants.

**Results:**

The number of first hospitalizations after age 70 is significantly higher at the HSI and NHI compared to NI. A significantly higher rate of suicide attempts was observed among HSI (13.8%), compared with the NI (11.8%) and NHI (9.7%). The odds for severe mental illness were significantly higher among HSI and NHI compared to NI by 84% and 66% among patients with psychotic disorders, twofold higher, and higher by 37% among patients with affective disorders, and threefold and 2.5 times higher among patients with anxiety.

**Conclusions:**

Exposure to the Holocaust trauma has an effect on patterns of psychiatric hospitalizations and the severity of Holocaust survivors’ psychiatric illness. Immigration is an independent risk factor for severe mental illness, although its influence was less pronounced than direct Holocaust exposure. Exposure to severe trauma such as war during childhood has long-term effects on the course and severity of mental illnesses.

## Introduction

Holocaust survivors were exposed to extreme catastrophic stress and trauma [1]. They experienced an ongoing threat to their lives, terrible living and working conditions, prolonged starvation, constant threats of death, and loss of family members, often before their very eyes. Some survivors were subjected to horrific medical experiments without their consent or knowledge [2], and others lived in hiding under the ever-present threat of discovery [3]. In the years that followed the Second World War (WWII), more than half a million Holocaust survivors immigrated to Israel, a state that was established as a homeland for Jews in 1948. In the years following the establishment of the state, Jews from around the world continued to immigrate to Israel, both as individuals and in organized groups. One of the most significant waves of immigration since the state’s founding was the influx of immigrants from the former Soviet Union (FSU) during the 1990s. This group stands out as the oldest immigrant cohort in Israel, with an average age at arrival of 54 years old [4]. Notably, the FSU immigration wave included a considerable number of older adults who were Holocaust survivors. Today, fewer than 150 thousand Holocaust survivors are still alive in Israel [5]. While their number is decreasing, it is crucial to keep studying the effect of extreme trauma on their lives. A group of survivors that is less studied consists of Holocaust survivors who were psychiatrically hospitalized during their lifetime. Very few reports of Holocaust survivors hospitalized in psychiatric wards were published in the nineteen sixties and seventies [6, 7].

It is assumed that surviving the Holocaust demanded significant resilience, which individuals with psychiatric disabilities would be less likely to possess, resulting in a very low survival rate for mentally ill persons during the Holocaust. Consequently, we assume that hospitalized survivors were people without serious psychopathology before the Nazi persecution that, following exposure to severe trauma, developed chronic mental pathology.

A variety of models suggest that severe enough negative events can precipitate disorders even without reference to individual biological or psychological characteristics [8]. Numerous works suggest that traumatic life events can negatively affect the course of schizophrenia and other severe mental illnesses (SMI) [9, 10], which is consistent with the stress-diathesis model [11, 12].

The model suggests that the likelihood of developing schizophrenia is a joint function of a genetic susceptibility expressed by hypersensitivity to stress and the actual environmental stress encountered. Norman and Malla reviewed 30 studies comparing the level of stress on the severity of symptoms of patients with schizophrenia. In 23 (77%) studies, there were statistically significant findings of life events with higher stress associated with more pronounced symptoms of the disorder [13].

The current study aims to examine whether the severity and characteristics of mental illness are different between Holocaust survivor immigrants (HSI), Native Israelis (NI), and Non-Holocaust Immigrants (NHI) (immigrants to Israel who were not exposed to the Holocaust).

According to the social defeat hypothesis [12], the negative experience of being excluded from the majority group may explain migration and childhood trauma as major risk factors for SMI. A consistent finding from epidemiological studies is the higher incidence rate of schizophrenia and other psychotic disorders among immigrants. In a meta-analysis of Selten and Cantor-Garaae (2005), a relative risk of 2.7 was estimated for first-generation immigrants compared with native populations [13]. For this reason, it is important to include the group of immigrants who were not exposed to the Holocaust, since immigration itself could be a confounding factor that needs to be controlled for.

## Methods

### Psychiatric case registry

Israel is one of the few countries globally that maintains a psychiatric case registry. The Israeli Psychiatric Case Registry (PCR) includes all psychiatric admissions in Israel since 1945 [14]. The registry is an important tool for health services planning in the field of mental health, for developing and accessing intervention programs and research. Institutions with psychiatric hospitalizations are required by law to file a report of each admission with the Ministry of Health. Ambulatory visits in any setting are not included in the Registry.

The demographic data submitted by the hospital for each patient is cross-referenced with information available from the Ministry of Interior’s population registry. This process is performed to enhance the accuracy of the data. The registry is regularly updated to include information on patient deaths, even when these occur many years after the last hospital discharge.

The Israeli psychiatric case registry has been using the *ICD-10* for its diagnostic classification since 1997 [14].

The following data were extracted from the case registry: place of birth, date of birth, gender, date of immigration, marital status, date of first admission and release, date of last admission and release, accumulated length of hospital stay, number of admissions, number of involuntary admissions, and diagnosis at release.

### Study population

We requested an unidentified list of hospitalized patients, according to the following criteria: Jewish patients who were born before 1944 and were admitted to a psychiatric ward between 1.1.1945 and 31.12.2009 (based on technical issues encountered with file creation, which prevented the inclusion of data beyond this point) while their age at first hospitalization was at least 18 years old and with the following ICD-10 diagnosis: schizophrenia and other functional psychoses (F20-F29), affective disorders (F30-F39), disorders due to alcohol and drug abuse (F10-F19), anxiety disorders (F40-F43), somatoform and dissociative disorders (F44-F49), personality disorders (F60-F62).

The current analysis was restricted to participants presumed to be of Ashkenazi Jewish origin (defined as individuals whose ancestry traces back to Central and Eastern European Jewish communities) in order to reduce cultural heterogeneity and enhance the internal validity of comparisons. As individual-level information on parental ethnic origin was not available, classification was based on country of birth. Participants born in countries where the Jewish population was historically predominantly Ashkenazi (e.g., Poland, Lithuania, Hungary, Germany, Czechoslovakia) were included in the analytic sample, while those born in countries with predominantly Sephardic (Jews descended from the historic Jewish communities of the Iberian Peninsula - Spain and Portugal - or their descendants who settled in North Africa, the Middle East, and other regions), or mixed Jewish populations were excluded. Following these exclusions, 30,539 records were selected from the original total of 48,533, and subsequently categorized into three groups:

1. **Holocaust Survivor Immigrants (HSI)**: Individuals of presumed Ashkenazi origin who were born before 1944 in a European country that was occupied or under the direct control of the Nazi regime and immigrated to Israel after the beginning of the Second World War (WWII) (i.e., after 1.9.1939). This is an accepted definition, used by Israel’s research and governmental institutes [14].
2. **Non-Holocaust Immigrants (NHI)**: Individuals who were born before 1944 in a European country and immigrated to Israel before the beginning of WWII. or were born in European countries that the Nazis did not occupy.
3. **Native Israelis (NI)**: Jewish individuals who were born before 1944 in what would after 1948 become Israel and raised in Israel.
4. The group of HSI includes 810 individuals who immigrated to Israel during the war. Immigration during WWII can imply different and maybe less severe exposure to the devastations of the Holocaust, but since no individual information regarding actual exposure to the Holocaust is available, this proxy variable was used.

### Construction of the holocaust impact variable

Among the group of Holocaust Survivor Immigrants (HSI) we constructed a contextual variable termed **Holocaust Impact** to assess the intensity of Holocaust-related trauma on a population level. This variable was designed to capture the relative degree of collective loss experienced by Jewish communities in different countries during the Holocaust. The assignment of Holocaust Impact was based on historical estimates of the proportion of the Jewish population murdered in each country under Nazi occupation or influence during World War II [15–25].

This continuous variable was further categorized into four levels (reduced: <30%, moderate: 30–59%, high: 60–84%, very high: 85–100%) to assess threshold effects (Table 1).

**Table 1:** Holocaust Impact among Holocaust Survivor Immigrants (n = 19770)

Country	*n*	%
**Reduced Impact countries < 30% Jews killed**		
**Total**	5751	100
Italy	61	1.1
Bulgaria	904	15.7
Former Soviet Union, excluding Ukraine, Belarus, Lithuania, Latvia, Estonia	4421	76.9
Russia	149	2.6
Denmark	6	0.1
France	210	3.7
**Moderate Impact countries = 30–59% Jews killed**		
**Total**	6397	100
Austria	121	1.7
Belgium	72	1.1
Norway	2	0.1
Romania	6202	97.0
**High impact countries = 60–84% Jews killed**		
**Total**	2373	100
Germany	345	14.5
Netherlands	68	2.9
Hungary	904	38.1
Yugoslavia	189	8.0
Latvia	151	6.4
Moldova	25	1.1
Czechoslovakia	691	29.1
**Very High Impact countries = 85–100% Jews killed**		
**Total**	5249	100
Ukraine	340	6.5
Belarus	67	1.3
Lithuania	59	1.1
Latvia	2	0
Greece	145	2.8
Poland	4637	88.3

### Statistical analysis

The follow-up time for Israeli-born participants was calculated from age 18 or the date of immigration, whichever occurred later. To define the outcome variable of Severe Mental Illness (SMI), we first calculated the proportion of follow-up time each participant spent hospitalized for psychiatric reasons. This was computed as:

Proportion of study time in hospitalization = Cumulative length of psychiatric hospitalization (days)/Total person time during follow-up (days).

This continuous variable was then dichotomized at the upper quartile; participants who spent 4% or more of their follow-up time in psychiatric hospitalization were classified as having SMI (outcome variable), while those below this threshold were not.

A Chi-square test was used to examine the association between the categorical variables, and the Kruskal-Wallis non-parametric test for continuous variables, which were not normally distributed.

Multivariate logistic regression analysis was performed to assess the probability of being at the upper quartile as to the proportion of study time in hospitalization (SMI).

As significant interaction was found between the study group and type of diagnosis (*p* <.0001) and between gender and type of diagnosis (*p* <.0001), this analysis was split by type of diagnosis, adjusted for gender, age at the beginning of follow-up, and age at death.

In addition, focusing only on Holocaust survivors, the association between the age at the beginning of the war and the probability of SMI was assessed with multivariable logistic regression, adjusted for diagnosis type, Holocaust Impact, age at the beginning of follow-up, and age at death. As a significant interaction was found between gender and age at the beginning of the war (*p* <.0001), this analysis was split by gender.

For all analyses performed, a value of *p* <.05 was considered statistically significant. Analyses were carried out with the SPSS version 25.0 statistical package (SPSS, Inc, Chicago, IL).

### Ethical approval

The study was reviewed and approved by the Herzog Memorial Hospital Ethics Committee. Written informed consent was not required in accordance with institutional and national policies. Analysis for this article was done on files that did not include the identity of the subjects.

## Results

### Study population characteristics

The demographic characteristics are depicted in Table 2. The proportion of females was highest among NHI (64.6%) in comparison with other study groups (61.8% among the HSI and 51.8% among the NI, *p* <.001). 

The frequency of widowed participants was highest among HSI (16.0%) vs. among NHI (13.2%) and NI (6.3%) (*p* <.001). At the date of file closure (31.12.2009) 20,934 of 30,539 patients perished (68.5%). 68.5% of the HSI group, 50.1% of the NI, and 88.7% of the NHI (*p* <.001). 

The average age of death was 71.2 years among HSI, 63.9 for the NI, and 74.4 for NHI (*p* <.001). 

The mean age of immigration was 36.0 years for HSI and 18.1 for NHI (*p* <.001). 40.2% of HSI were adults at the beginning of WWII compared to 11.2% of NI and 78.7% of NHI.

The hospitalization characteristics of the study population are represented in Table 3. HSI and NHI patients were significantly older at their first hospitalization (mean age = 53.7 and 55.7 years vs. 40.7 among the NI), 18.2% of the HSI and 19% of NHI were first hospitalized after the age of 70, compared with only 5.4% of the NI. In all three groups, the most common diagnoses among those who were first hospitalized after age 70 are affective disorders 61.2%, psychotic disorders 27.2%, and anxiety disorders 7.8% (data is not presented). A significantly higher rate of suicide attempts was observed among HSI (13.8%), compared with the NI (11.8%) and NHI (9.7%). In the group aged 70 + at first hospitalization, the suicide rate of HSI was significantly higher (21.0%) than among NI (15.0%) and NHS (19.7%) (*p* <.001)(data is not presented).

**Table 2:** Demographic characteristics and mortality of the study population

Characteristics	Holocaust Survivors Immigrants (HSI)	Native Israelis (NI)	Non-Holocaust Immigrants (NHI)	Total
	*n* = 19,770	*n* = 5,616	*n* = 5,153	*n* = 30,539
Gender** (% of females)	61.8	51.8	64.6	60.5
Marital status (%)**				
Single	5.8	9.8	3.7	6.2
Married	32.2	33.9	26.7	31.6
Divorced	7.6	9.4	3.5	7.3
Widowed	16.0	6.3	13.2	13.7
Missing	38.3	40.6	53.0	41.2
Age at the beginning of the war** (01.09.1939), years				
< 2 years	11.8	38.0	0.3	14.7
2–5 years	8.8	19.7	1.3	9.5
6–11 years	17.1	18.4	6.8	15.6
12–14 years	10.4	7.0	4.6	8.8
15–17 years	11.7	5.2	8.3	9.9
18 + years	40.2	11.7	78.7	41.5
Age at immigration, years**				
Mean (SD)	36.0 (16.4)	-	18.1 (8.4)	32.3 (16.8)
Median (IQR)	34.0 [23.0–47.0]	-	19 [13.0–23.0]	29.0 [20.0–43.0]
Death rate**	68.5	50.1	88.7	68.5
Age at death, years				
Mean (SD)	71.2 (11.4)	63.9 (13.0)	74.4 (10.8)	70.9 (11.9)
Median [IQR]	72.0 [64.0–79.0]	65.0 [56.0–73.0]	75.0 [68.0–82.0]	72.0 [64.0–79.0]

** *p* <.001

**Table 3:** Hospitalization characteristics of the study population

Hospitalization characteristics		HSI	NI	NHI	Total
Age at first admission**	Mean (SD)	53.7 (16.1)	40.7 (16.1)	55.7 (14.7)	51.7 (16.7)
	Median [IQR]	54.0 [42.0–66.0]	38.0 [27.0–53.0]	46.0 [34.0–59.0]	52.0 [39.0–65.0]
Length of 1 st admission in days**	Mean (SD)	183.1 (844.5)	200.5 (999.3)	253.2 (1171.9)	198.2 (936.8)
	Median [IQR]	44.0 [20.0–90.0]	50.0 [21.0-101.0]	51.0 [26.0-101.0]	46.0 [21.0–94.0]
Length of the last admission in days**	Mean (SD)	431.2 (1484.6)	525.9 (1859.3)	563.6 (1779.2)	471.0 (1612.2)
	Median [IQR]	44.0 [20.0-103.0]	46.0 [20.0-104.0]	56.0 [27.0-129.0]	46.0 [21.0-107.0]
Number of admissions**	Mean (SD)	4.0 (5.2)	5.2 (7.2)	4.0 (5.1)	4.2 (5.6)
	Median [IQR]	2.0 [1.0–5.0]	3.0 [1.0–6.0]	2.0 [1.0–5.0]	2.0 [1.0–5.0]
Rate of first admission above the age of 70** (%)		18.2%	5.4%	19.0%	16.0%
Suicide attempts** n (%)		2730 (13.8)	662 (11.8)	499 (9.7)	3891 (12.7)
Compulsory hospitalization** n (%)		3402 (17.2)	1165 (20.7)	464 (9.0)	5031 (16.5)

**p of Kruskal-Wallis non-parametric test < 0.001

### Mental illness types among the study population

 Psychotic disorders are more common among the NI group (49.1%) compared to 42.2% among HSI and 36.1% among NHI. The proportion of patients with affective disorders was the highest among NHI (54.0% vs. 33.7%−45.1%).(p<.001) (Table 4).

**Table 4:** Mental illness diagnoses in the study population

	Mental illness diagnosis						
	Psychotic disorders	Affective disorders	Anxiety disorders	Alcohol & drug-related	Somatoform & dissociative	Personality Disorders	Total
HSI n (%)	8349 (42.2%)	8917 (45.1%)	1202 (6.1%)	616 (3.1%)	315 (1.6%)	371 (1.9%)	19770
NI n (%)	2757 (49.1%)	1891 (33.7%)	446 (7.9%)	182 (3.2%)	79 (1.4%)	261 (4.6%)	5616
NHI n (%)	1858 (36.1%)	2781 (54.0%)	251 (4.9%)	92 (1.8%)	78 (1.5%)	93 (1.8%)	5153

*p* of χ² test < 0.001

### The probability of severe illness, univariate and multivariable analysis in total study population

**Fig. 1:**
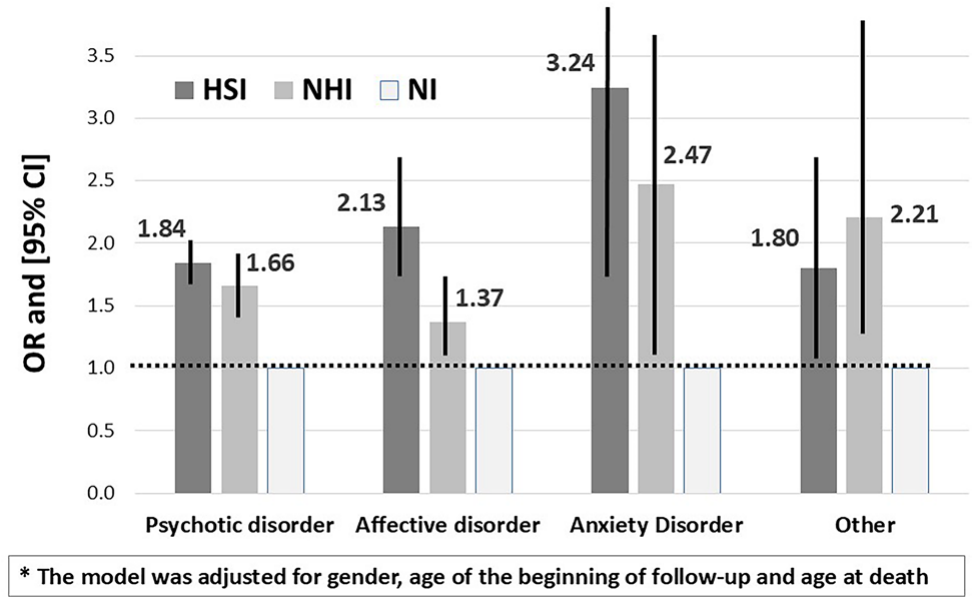
The probability of Severe Mental Illness (SMI): multivariable Logistic regression model*, by type of mental illness

Since there was a statistically significant interaction between diagnosis and gender (*p* <.0001) and between diagnosis and the HSI group (*p* <.0001), the analysis was performed separately in every diagnostic group. The results of the multivariable analysis with SMI as the dependent variable are depicted in Figure 1. The odds for severe illness were significantly higher among HSI and NHI compared to NI by 84% and 66% among patients with psychotic disorders (OR=1.84, 95% CI:1.62-2.10 and 1.66, 95% CI: 1.42-1.94, respectively), twofold higher and higher by 37% among patients with affective disorders (OR=2.13, 95% CI: 1.73-2.62 and OR=1.37, 95% CI: 1.08-1.72, respectively) and threefold and 2.5 higher among patients with anxiety (OR=3.24, 95% CI: 1.83-5.74 and OR=2.47, 95% CI: 1.18-5.16, respectively). The significantly higher odds of severe illness among HSI and NHI vs. NI were also found among patients with other disorders.

Among patients with psychotic disorders, males exhibited significantly higher odds of experiencing severe illness (OR=1.11, 95% CI: 1.01-1.21). Among patients with affective disorders, males exhibited significantly lower odds of experiencing severe illness (OR=0.67, 95% CI: 0.60-0.76) (data is not presented in Figure 1).

### The probability of severe illness, univariate and multivariable analysis in holocaust survivors

Additional analysis was conducted only among Holocaust survivors, males, and females separately (Table 5), as a significant interaction was found between the gender and age at the beginning of the war. Having psychotic disorder (vs. other) was associated with significantly higher odds for SMI as defined by our research, both among males (OR = 5.8, 95% CI: 4.7–7.2) and females (OR = 6.1, 95% CI: 4.5–8.3). Having an affective disorder was associated with significantly higher odds, but this association was observed only among females (OR = 2.1, 95% CI: 1.5–2.8).

**Table 5:** The multivariable logistic regression model for the probability of SMI among Immigrant Holocaust Survivors

Characteristics	Immigrant Holocaust Survivors	SMI (Severe Mental Illness)	Multivariable Analysis	
			Odds Ratio (OR) [95% CI]	
	*n* (%)	*n* (%)	Model I (Males)	Model II (Females)
Total Population	19,770 (100)	5,239 (26.5)		
Gender				
Males	7,546 (38.2)	2,079 (27.6)	-	-
Females	12,224 (61.8)	3,160 (25.9)	-	-
Mental illness				
Psychotic disorder	8,349 (42.2)	3,490 (41.8)	**5.8** **(4.7–7.2)**	**6.1** **(4.5–8.3)**
Affective disorder	8,917 (45,1)	1,453 (16.3)	1.1 (0.9–1.4)	**2.1** **(1.5–2.8)**
Anxiety disorder	1,202 (6.1)	117 (9.7)	0.9 (0.6–1.3)	1.1 (0.7–1.7)
Other* disorders	1,302 (6.6)	179 (13.7)	reference group	
Age at the beginning of the war (01.09.1939)				
< 2 years	2,334 (11.8)	578 (24.8)	reference group	
2–5 years	1,736 (8.8)	479 (27.6)	**1.5** **(1.1–2.0)**	0.9 (0.6–1.2)
6–11 years	3,387 (17.1)	878 (25.9)	**1.6** **(1.2–2.1)**	1.1 (0.8–1.4)
12–14 years	2,059 (10.4)	470 (22.8)	**1.5** **(1.2–2.1)**	1.1 (0.8–1.4)
15–17 years	2,305 (11.7)	578 (25.1)	**1.5** **(1.1–2.0)**	1.1 (0.9–1.5)
18 + years	7,949 (40.2)	2,256 (28.4)	**1.4** **(1.1–1.8)**	**1.5** **(1.1–1.9)**
Holocaust Impact				
Reduced Impact	5,751 (29.1)	1,460 (25.4)	reference group	
Moderate Impact	6,397 (32.4)	1,802 (28.2)	**1.2** **(1.1–1.4)**	1.1 (0.9–1.2)
High Impact	2,373 (12.0)	630 (26.5)	1.2 (0.9–1.5)	1.0 (0.9–1.2)
Very High Impact	5,249 (26.6)	1,347 (26.5)	1.1 (0.9–1.3)	0.9 (0.9–1.1)

* “Other” includes alcohol & drug-related, somatoform & dissociative and personality disorders

**Model I (among males) and Model II (among females)** includes age at the beginning of war, of mental illness and Holocaust impact

Statistically significant values are shown in bold

Being older at the beginning of the war was associated with an increase in the odds of severe illness. Thus, those participants who were 18 years old and older had 40% and 50% higher odds for SMI (OR = 1.4 and OR = 1.5 for males and for females, respectively) in comparison with those who were young children less than 2 years old.

Being male, exposed to the moderate (vs. reduced) Holocaust Impact, was associated with significantly higher odds for SMI (OR = 1.2, 95% CI: 1.1–1.4). 

## Discussion

The current study aimed to assess the differences in illness characteristics and severity between psychiatrically diagnosed HSI, NI, and NHI patients. Our study revealed that Holocaust survivors had higher odds of severe illness in affective, psychotic, and anxiety disorders (in comparison with NI and NHI). Additionally, immigration itself emerged as an independent risk factor for severe mental illness (SMI), as indicated by increased odds for SMI across all diagnoses in NHI. The risk associated with immigration was lower than that observed for the Holocaust Impact. This finding is consistent with the numerous studies that found a high prevalence of trauma and PTSD amongst individuals with SMI, relative to the general population [8, 26–28].

Furthermore, trauma was associated with adverse psychosocial functioning, increased symptom severity, and increased healthcare burden. Ross Anderson and Clark (1994) showed that patients diagnosed with schizophrenia who presented with a history of physical and sexual abuse in childhood were more likely to report positive symptoms as well as more severe symptoms overall. Patients without such a history had predominantly negative symptoms [29]. 56.9% of HSI with SMI in our study were children at the beginning of WWII. Recent studies have shown that childhood trauma increases the likelihood of developing severe mental disorders [30] and is associated with slower improvement rates [31].

This is conceptually consistent with the diathesis-stress model, which posits that mental illnesses such as psychotic and affective disorders have both biological and environmental components [32]. The experience of trauma is a psychosocial stressor that contributes to higher levels of symptoms and, therefore, to longer hospitalization. Terano et al (1998) reported on 44 patients at a psychogeriatric department in Israel and a control group of patients born in Israel. Lengths of hospitalization, the number of previous hospitalizations, and the cumulative length of hospitalization were compared. All indices were higher in Holocaust survivors [33].

Having an affective disorder was associated with significantly higher odds for SMI, only among females (OR=2.1, 95% CI: 1.5-2.8). This finding is consistent with previous studies showing that women are more likely to develop post-traumatic stress disorder (PTSD) and other stress-related mental disorders following exposure to a traumatic event [34]. Although findings pertaining to gender phenomenology among Holocaust survivors have varied [35], there are reports of increased levels of emotional distress found among women survivors compared with male survivors and the control group of non-survivors [36]. Our results regarding older age of death in HSI and NHI compared to NI (71.2, 74.4, and 63.9, respectively), correspond to previous findings such as Fund et al, indicating that the mean age of death in Holocaust survivors was significantly higher than that of a matched Israeli-born control group. In contrast, their physical health was poorer, indicated by a higher rate of physical comorbidities [37]. Similarly, in another study, Shrira et al. also found that concentration camp survivors exhibited a mortality risk that was either lower or comparable to that of the other groups, while functioning in psychosocial markers was lower [38]. Sagi-Schwartz et al. reported on the relative longevity of Holocaust survivors compared to non-Holocaust survivors and suggested that individuals who managed to survive the Holocaust might have exhibited greater resilience due to possessing more advantageous genetic, physical, and emotional attributes [39]. These studies were conducted on the general population, while our research is the first to report the same results when examining the population of psychiatrically hospitalized patients. In our study, the death rate was significantly higher in NHI (88.7) compared to HSI (68.5) and NI (50.1). Youssim et al. (2021) found higher mortality among survivors: all-cause and cancer-specific mortality in women, as well as cancer-specific and coronary heart disease-specific mortality in men [40].

A remarkable finding was the fact that the first hospitalization above the age of 70 was considerably more frequent among the HSI and NHI than in the NI. Most were hospitalized with affective disorders. Immigration itself appears to act as a sustained risk factor, as evidenced by studies showing elevated psychiatric morbidity among immigrants from FSU compared to native-born populations, even after accounting for Holocaust-specific trauma [41]. Delayed help-seeking behaviors, particularly prevalent among immigrant populations due to cultural stigma and distrust of institutional healthcare systems, may exacerbate untreated mental health conditions until crisis points requiring hospitalization [42]. Additionally, social fragmentation in aging immigrant populations - characterized by diminished family networks and community support - contrasts with the stronger intergenerational ties typically observed in Israeli-born elderly, potentially delaying early interventions [42]. These findings align with broader patterns of immigrant mental health disparities, where migration-related stressors interact with aging processes to amplify psychiatric risks [41].

A poorer mechanism of coping may also explain the findings of Barak et al. They collected 374 files from psychiatric hospitalizations of older adult Holocaust survivors in Israel and found that they had an increased risk of attempting suicide compared to non-Holocaust survivors [43]. Our findings regarding suicide attempts align with this study revealing an increase in suicide attempts among HSI but especially above age 70, compared with the other study groups. Another reason for the increased suicide attempts in older adult HSI may be reduced familial support, as many of these seniors have experienced significant losses of family members throughout the course of the war. This also aligns with our findings of increased widowhood among HSI compared to NI and NHI. Tendency to suicide accompanying severe mental conditions and lack of effective coping, while the patient ages, was previously documented among older adult Holocaust survivors [44]. In contrast to that, Lurie et al. did not find a greater risk of suicide in Holocaust survivors than in a comparison group [45]. A further study showed a significantly higher suicide risk for persons who immigrated to Israel from the beginning of Nazi persecution but before the beginning of extermination, compared to HSI [46].

Age at the beginning of War (age at the start of traumatic exposure) was found to be associated in the current study with the probability of developing SMI in every type of mental diagnosis, while aged 18+ were found to have higher odds for SMI than younger children.

Analysis of age distribution within the non-immigrant (NI) group reveals that the majority were infants (aged 0–2 years) during the Holocaust. Consequently, during the 1948 Israeli War of Independence - occurring nine years later - this cohort was aged 9-11 years, with 57.7% exposed to war-related trauma during childhood (age 0-14). This finding raises critical questions about the long-term psychiatric consequences of early-life exposure to armed conflict, independent of Holocaust-specific experiences. For instance, Akbulut-Yuksel et al. (2022) demonstrated that German children exposed to WWII bombings before age five faced a 16.2% increased risk of late-life clinical depression [47].

While Holocaust survivors endured multi-layered trauma (genocide, displacement, and acculturative stress), the Israeli-born cohort’s psychiatric hospitalizations as adults may reflect delayed manifestations of childhood war exposure, as seen in non-Holocaust populations exposed to early-life violence [31, 46, 48]. The heightened odds of severe mental illness (SMI) in Holocaust survivors compared to the NI group likely stem from the compounding effects of genocide, migration, and aging-related stressors. These findings underscore the urgency of implementing early interventions for children exposed to conflict, as advocated by the WHO [49], to mitigate lifelong psychiatric risks [50]. The lower rate of mental hospitalizations among child Holocaust survivors in adulthood compered to adult survivors does not necessarily indicate an absence of trauma effects, rather, it may reflect the fact that most children who were exposed to the most extreme trauma did not survive, and those who did were likely to possess unique protective characteristics. 

### **Strength and limitations**

The main strength of this study is the large national sample size. Nevertheless, in the current database, the information regarding personal exposure to the Holocaust of each patient does not exist, and therefore the presented data is estimated according to year of birth, country of birth, and date of immigration to Israel. The Holocaust's impact was determined based on country of birth, which may not accurately reflect an individual’s actual location during WWII, as some individuals may have fled during the Holocaust period or relocated from their country of origin.

Our database lacks Socio-Economic Status (SES), while SES may be related to resources that prevent a patient`s mental deterioration. Due to a lack of data on the exact type of exposure to the Holocaust, we treated Holocaust survivors as a homogeneous group, although clearly, the exposure to trauma was different between survivors, according to their presence in Ghettos, labor camps, concentration camps, hiding, etc.

To calculate the prevalence of the different mental disorders, the precise numbers of total immigrants and native Israelis and their birth dates are needed. Since this data wasn’t available, it was impossible to compare the prevalence of the various disorders among the three groups.

The policy regarding psychiatric hospitalization underwent changes over the years. However, it seems unlikely that these changes were solely focused on Holocaust survivors, and so it should not be expected to influence the distinctions between the groups significantly.

 For the immigrants, there could be potentially unaccounted hospitalizations in their country of origin.

Finally, the data was restricted to the end of 2009. As a result, any trends or changes in mental illness characteristics and severity that occurred after 2010 are not captured in this analysis. On the other hand, since after 2010, the subjects in the cohort are aged 65 and above, it is not expected that expanding the cohort would have significantly changed the results. This is because psychiatric hospitalizations in older adults can often be related to secondary factors such as medical conditions, medications, or cognitive impairments like dementia [51, 52].

## **Conclusions**

In the population of mentally hospitalized patients, both Holocaust Survivor Immigrants and Non-Holocaust Immigrants had an increased risk of having a severe mental disorder as compared to the non-immigrant population.

 Exposure to the holocaust trauma and immigration can manifest many years later, often becoming apparent in old age, as indicated by our findings of initial hospitalizations occurring at this stage of life. Early identification and targeted mental health support for individuals exposed to trauma in childhood or who have experienced migration are crucial, even decades after the traumatic event. The findings underscore the need for mental health policies tailored to the unique histories and vulnerabilities of immigrants and trauma survivors. 

## Data Availability

No datasets were generated or analysed during the current study.
